# Comprehensive Analysis of the Expression and Prognostic Value of LMAN2 in HER2+ Breast Cancer

**DOI:** 10.1155/2022/7623654

**Published:** 2022-06-06

**Authors:** Di Zhang, Liping Ye, Shuang Hu, Qingqing Zhu, Chenxi Li, Chengming Zhu

**Affiliations:** Scientific Research Center, The Seventh Affiliated Hospital, Sun Yat-sen University, Shenzhen, Guangdong 518107, China

## Abstract

Lectin, Mannose Binding 2 (LMAN2) encodes a type I transmembrane lectin that shuttles between the plasma membrane, the Golgi apparatus, and the endoplasmic reticulum. However, its expression, prognosis, and function in invasive breast carcinoma remain unknown. Nine databases were consulted to evaluate LMAN2 expression and prognosis in breast cancer. The possible function of LMAN2 in breast cancer was investigated in the Human Cell Landscape (HCL) database, Gene Regulatory Network database (GRNdb), and CancerSEA database. Moreover, N6-methyladenosine (m6A) modifications were analyzed using the RMBase v2.0 and M6A2Target databases. Seven databases were then used to analyze the potential action mechanisms of LMAN2. Our findings suggest that LMAN2, which is expressed at a high level in breast cancer, is linked to an unfavorable prognosis. Therefore, LMAN2 has the potential to be utilized as a treatment target in breast cancer. Furthermore, the single-cell analysis illustrated that LMAN2 expression had a positive link to breast cancer stemness, proliferation, metastasis, and differentiation. Moreover, m6A modifications were found in the LMAN2 gene. Consequently, modifications to m6A methylation may influence LMAN2 expression, which is associated with the homologous recombination (HR) in its DNA damage repair pathway .

## 1. Introduction

Breast cancer has a remarkably high incidence rate and is the second major contributor to cancer deaths among women [1]. Breast cancer may be grouped into four kinds premised on their molecular subtypes: HER2-positive breast cancer (HER2+, ER–, and PR–);basal-like (ER–, PR–, and HER2–);luminal B (ER+, PR+, HER2–, and Ki67 ≥ 14% or ER+, PR+, and HER2+); and luminal A (estrogen receptor [ER] +, progesterone receptor [PR] +, HER2–, and Ki67 (proliferation marker < 14%)). HER2-positive breast cancer is found in 20–30% of patients with breast cancer. It is an aggressive high-grade cancer that is negative for ER and PR [2]. Moreover, HER2-positive breast cancer is amenable to a comprehensive treatment plan of chemotherapy combined with Herceptin. However, the prognosis of HER2-positive breast cancer is worse than that of luminal A and B cancers. HER2-positive patients tend to be younger and have more rapid disease progression, larger tumors, higher histological grades, and more lymph node metastases. In addition to targeted drugs, treatment of advanced HER2-positive breast cancer patients mainly involves a blend of endocrine, chemotherapy, and immunotherapy; however, chemotherapy or endocrine resistance is inevitable treatment [3–6]. The breast cancer cells' capacity to withstand pharmacological treatment has been shown in many studies [7–9]. Therefore, searching for potential HER2-positive breast cancer target molecules can help overcome the threat posed by drug resistance. With the development of molecular biology techniques, integrated analysis of multiple omics databases has facilitated the identification of molecular targets and biomarkers in cancer.

LMAN2, a protein-coding gene, is responsible for encoding a type I transmembrane lectin that shuttles between the plasma membrane, Golgi apparatus, and endoplasmic reticulum. Glycoproteins with a high mannose content are bound by the encoded protein, which may aid in sorting glycoproteins, their transportation, and quality assurance. LMAN2 is involved in biological processes such as protein metabolism, Golgi transport dynamics, and subsequent modifications. LMAN2 is a candidate tumor biomarker for intraperitoneal chemotherapy in the treatment of ovarian cancer [10]. LMAN2 regulates the transport of exosomal cargo proteins through the Golgi complex [11].

In this research, we utilized multiple online databases to examine the prognostic impact of LMAN2 and found that the elevated LMAN2 expression is linked to the unsatisfactory prognosis of HER2-positive breast cancer. We used Gene Expression Profiling Interactive Analysis (GEPIA), Breast Cancer Gene-Expression Miner v4.7 (bc-GenExMiner v4.7), UALCAN, The Human Protein Atlas (HPA), Gene Expression-Based Outcome for Breast Cancer Online (GOBO), Cancer Cell Line Encyclopedia (CCLE), SpatialDB, and Tumor Immune Estimation Resource (TIMER) databases to evaluate the LMAN2 expression. To additionally investigate the LMAN2 expression and its impact on patients' prognoses, we utilized the bc-GenExMiner v4.7 and Kaplan–Meier Plotter databases. Moreover, we employed M6A2Target, RMbase, and sequence-based RNA adenosine methylation site predictor (SRAMP) databases to analyze LMAN2 RNA methylation levels and prognosis. Next, R v4.0.3 was utilized to examine the differences in the expression levels of DNA damage repair HR protein in HER2-positive breast cancer samples from The Cancer Genome Atlas (TCGA) database and construct a prognostic DNA damage repair prognostic model for LMAN2 and DNA damage repair HR protein in HER2 subtypes. Databases such as the HCL database and CancerSEA were used to further analyze the feature map of LMAN2 and its correlation with the functional status of breast cancer samples at the single-cell level. Then, the link between the expression level of LMAN2 and drug sensitivity GDSC (Genomics of Drug Sensitivity in Cancer) was analyzed. Eventually, we explored possible mechanisms of action of LMAN2 using Gene Ontology (GO), Gene Set Enrichment Analysis (GSEA), STRING, GRNdb, GeneMANIA, and Domain Interaction Graph Guided ExploreR (DIGGER). In summary, a comprehensive analysis of the above-mentioned databases shows that LMAN2 expression is affected by its m6A methylation status and that LMAN2 alters the DNA damage repair pathway, thereby affecting the prognosis of HER2-positive breast cancer patients.

## 2. Methods

### 2.1. bc-GenExMiner v4.7

We used the bc-GenExMiner v4.7 database (http://bcgenex.ico.unicancer.fr) to examine the expression and prognostic significance of the LMAN2 gene in breast cancer. bc-GenExMiner v4.7 is a statistical processing platform for processing annotated breast cancer transcriptome data (DNA microarrays (*n* = 11,359)) that have been published and RNA-Seq (*n* = 4,712) [12, 13]. The LMAN2 expression and its correlation with cancer prognosis were analyzed in distinct types of breast cancer. In the present analysis, a published annotated breast cancer transcriptomic dataset was used (all DNA microarray data (*n* = 10,644)). Exhaustive prognostic analysis was conducted for LMAN2 with any nodal, ER, and PR status and data on distant metastasis-free survival (DMFS), recurrence-free survival (RFS), and overall survival (OS) (*n* = 9,422). The parameters for analysis were set as follows: *p* ≤ 0.05 and hazard ratio (HR) (values are rounded to two decimal places).

### 2.2. GOBO

To explore the expression levels of LMAN2, the GOBO database (http://co.bmc.lu.se/gobo/) was used. GOBO facilitates the exploration of gene expression profiles in breast cancer subtypes and breast tumor cell lines for gene sets, and also, the discovery of candidate metagenes premised on iterative correlation analysis to a prototype gene [14]. Only analyses concerning LMAN2 expression in breast cancer cells and their prognostic significance in all tumor types (*n* = 1,881) were examined. The correlation between LMAN2 expression and DMFS, RFS, and OS was also examined. Values with *p* < 0.05 were determined as having a statistical significance.

### 2.3. GEPIA

To study the expression levels and prognostic features of the LMAN2 gene, we employed the GEPIA database (http://gepia.cancer-pku.cn/). GEPIA is an online platform that utilizes a standard processing pipeline for scrutinizing the RNA sequencing (RNA-Seq) data of 9,736 tumors and 8,587 normal samples from the Genotype-Tissue Expression (GTEx) and TCGA databases [15]. The LMAN2 expression in breast cancer (*n* = 1,085) and normal samples (*n* = 291) was investigated in this work. Values with *p* < 0.05 were determined as having a statistical significance.

### 2.4. UALCAN

To identify the expression levels of LMAN2 and its correlation with relevant clinical features, we employed the UALCAN database (http://ualcan.path.uab.edu/index.html). UALCAN facilitates the analysis of miRNA gene expression using TCGA datasets and protein expression utilizing data from the Clinical Proteomic Tumor Analysis Consortium (CPTAC) Confirmatory/Discovery dataset [16]. Clinical features such as nodal status, race, sex, subclasses, menopause status, sample types, age, and tumor stage were assessed using multivariate parameters. In this study, the correlation between LMAN2 expression with multiple clinicopathological factors, such as ethnicity, sex, subclass, and age stratification, was analyzed in breast tumor and normal samples. The parameters for analysis were set as indicated: *p* value: 0.05, ^∗∗∗^*p* ≤ 0.001; ^∗∗^*p* ≤ 0.01; ^∗^*p* ≤ 0.05; ns. *p* > 0.05.

### 2.5. Kaplan–Meier Plotter

Utilizing Kaplan–Meier (KM) plotter database, we assessed if LMAN2 is a possible survival indicator for breast cancer. The KM plotter can be utilized to examine the impact of 54,000 genes (protein, miRNA, and mRNA) on survival in 21 distinct kinds of cancers [17]. To examine the link between LMAN2 (mRNA) on survival in breast cancer (*n* = 7,830) and its clinical significance, multivariate parameters were restricted to the analysis of subtypes and clinical significance. Examples of these parameters include the following:

- ER status-array: *n* = 7535
- PR status-IHC: *n* = 3548
- Human epidermal growth factor receptor 2 (HER2) status-array: *n* = 7535
- TP53 status: *n* = 660
- Subtype St Gallen: *n* = 7535
- Lymph node status: *n* = 4994; grade: *n* = 4429
- Subtype-PAM50: *n* = 7535

### 2.6. STRING

The interactions between LMAN2 proteins were investigated with the help of the STRING database (https://string-db.org/). Through the combination of known and anticipated protein-protein interaction (PPI) data for many organisms, the STRING database is aimed at collecting and integrating this information [18]. The link between the expression of LMAN2 and its cointeracting proteins was analyzed in this work. The links in STRING encompass both indirect (functional) and direct (physical) interactions, provided both are specific and biologically relevant.

### 2.7. HPA

The expression of the LMAN2 protein was examined utilizing the HPA database (http://www.proteinatlas.org/). The HPA is a Swedish-based project that started in 2003 with the primary focus being mapping all human proteins in organs, tissues, and cells premised on combined omics technological systems, such as mass spectrometry-based proteomics, transcriptomics, antibody-based imaging, and system biology [19]. Immunofluorescent cells and histopathological sections are analyzed for the spatial location and expression of LMAN2 expression in breast cancer. In the present investigation, the LMAN2 protein expression was analyzed in breast cancer and normal tissues.

### 2.8. CancerSEA

We examined the link between the LMAN2 of functional states in various single-cell datasets in breast cancer with the help of the CancerSEA database (http://biocc.hrbmu.edu.cn/CancerSEA/). CancerSEA is the very first specialized repository to thoroughly decode different functional statuses of cancer cells at the single-cell level [20]. In this project, we analyzed the correlation of LMAN2 with DNA damage repair, inflammation, metastasis, and differentiation. The parameters for analysis were set with the following values:

- Filter by correlation strength: 0.3
- Filter by *p* value: 0.05, ^∗∗∗^*p* ≤ 0.001; ^∗∗^*p* ≤ 0.01; ^∗^*p* ≤ 0.05; ns. *p* > 0.05
- Grey points were not considered when computing the correlations

### 2.9. HCL

Single-cell RNA sequencing data were utilized to assess the cell-type composition of key human organs and develop a basic scheme for HCL in order to investigate the feature map of the LMAN2 gene in breast cancer samples and the location of the LMAN2 gene in the marker gene table [21]. The tSNE map for breast-epithelium-Nguyen uses the human cell landscape database (http://bis.zju.edu.cn/HCL/). We downloaded the breast cancer single-cell LMAN2 gene expression (DGE) matrix through the gallery module and obtain the number of cells in the data and the sample source information. We used marker genes to analyze the results of LMAN2 gene clustering. As a result of our search for LMAN2 in the breast-epithelium-Nguyen, we received the following information: bar chart, feature plot, and the LMAN2's position in the marker gene table. A *p* value < 0.05 was selected as the criterion for statistical significance.

### 2.10. TIMER

To investigate the infiltration status of immune cells and the expression of LMAN2 in tumors, we retrieved the TIMER database (https://cistrome.shinyapps.io/timer/). TIMER is a powerful platform for the systematic investigation of immune infiltrates in cancers of a variety of different kinds of tumors [22–26]. In the present research, the LMAN2 expression was analyzed in multiple tumor and adjacent nontumor samples. Values with *p* < 0.05 were determined as having a statistical significance.

### 2.11. GDSC

To predict each sample's responsiveness to chemotherapy premised on one of the most comprehensive freely accessible pharmacogenomics GDSC (https://www.cancerrxgene.org/) database [27–29], tumor RNA-Seq data (FPKM) was acquired from the Genomic Data Commons (GDC), PFKM data were converted to TPM, and the log2 data (TPM + 1) were normalized, whereas recording the clinical information for each sample. With the aid of the R package “pRRophetic,” the prediction procedure was carried out by estimating the sample's half-maximal inhibitory concentration (IC50) by ridge regression and then calculating the predictive performance for each sample. All parameters were adjusted to their default settings after the batch effect of combat and tissue types of all solid tumors were removed, and the expression level of duplicated genes was presented as the mean score. In this research, the association of LMAN2 with multiple drugs was analyzed. All analyses were conducted using R packages provided by the R Foundation for Statistical Computing (2020), version 4.0.3.

### 2.12. GSEA and GO

Next, the enrichment of LMAN2 in GO and GSEA was analyzed. GSEA derives its strength from an emphasis on gene sets, which are groups of genes that have similar biological functions, chromosomal placement, or regulatory mechanisms. We employed the Enrichr database (http://amp.pharm.mssm.edu/Enrichr) to conduct GO annotation analyses to reveal the functions of LMAN2 [30]. The GO terms included cellular component (CC) and biological process (BP). Adj. *p* ≤ 0.05.

### 2.13. CCLE

The breast tumor-related cell line expression matrix was derived from the CCLE dataset (https://portals.broadinstitute.org/ccle/about). Multiple breast tumor-related cell lines were utilized in this investigation to examine the LMAN2 expression. The abovementioned analysis was conducted with the help of the R v4.0.3 software package ggplot2 (v3.3.3) [31].

### 2.14. SpatialDB

To explore the interaction relationship between LMAN2 proteins, the SpatialDB database (http://www.spatialomics.org/SpatialDB/) was used. In this study, the spatially resolved transcriptome of LMAN2 was analyzed in breast cancer. SpatialDB is the first online platform dedicated to curating geographically resolved transcriptome data from peer-reviewed publications, with the goal of providing a complete and reliable resource of spatial gene expression patterns in tissues [32].

### 2.15. GRNdb

To explore the interaction between LMAN2 proteins, the GRNdb database (http://www.grndb.com/) was used. In this study, the transcription factors (TFs) that regulate LMAN2 in breast cancer were identified. GRNdb is a publically available and user-friendly repository that enables easy exploration and visualization of anticipated modulatory networks generated by transcription factors (TFs) and their downstream target genes (colloquially known as regulons) premised on large-scale RNA-Seq data and also recognized TF-target associations in a variety of human and mouse disease models [33, 34].

### 2.16. GeneMANIA

To explore the interaction between LMAN2 proteins, the GeneMANIA database (http://genemania.org/) was also used. GeneMANIA anticipates the functions of genes from the composite network utilizing a variant of the Gaussian field label propagation technique that is optimized for predicting gene function, which often has a small number of positive examples [35]. The present work examined the interaction of LMAN2 with different molecules.

### 2.17. DIGGER

To explore the interaction between LMAN2 proteins, the DIGGER database (https://exbio.wzw.tum.de/digger/) was used. DIGGER maps interaction residues to exons by combining protein-protein and domain-domain interactions into a converged network [36]. The present investigation examined the interaction of LMAN2 with different molecules.

## 3. Results

### 3.1. LMAN2 is highly expressed in HER2+ breast cancer

**Figure 1:**
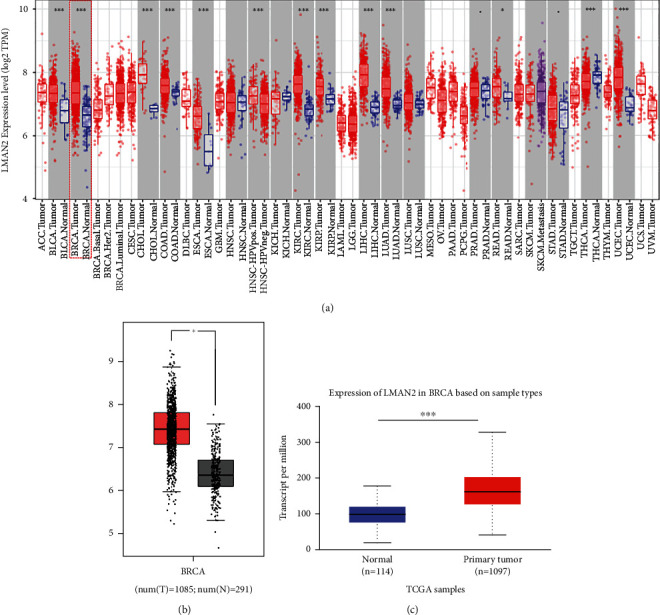
High expression level of LMAN in breast cancer cells. (a) The TIMER database was utilized to evaluate the LMAN2 expression in various malignancies. (b) The LMAN2 expression in normal and primary breast cancer tissues premised on the GEPIA database. (c) The expression of LMAN2 in normal and primary breast cancer tissues based on the UALCAN database (^∗∗∗^*p* ≤ 0.001; ^∗∗^*p* ≤ 0.01; ^∗^*p* ≤ 0.05).

Utilizing the TIMER database, we examined the expression of LMAN2 in tumor samples and nearby normal samples from various kinds of cancer. LMAN2 mRNA expression levels were remarkably increased in cancer tissues as opposed to matching normal samples (*p* < 0.05) (Figure 1(a)). With the use of the GEPIA and UALCAN database systems, we were able to determine the LMAN2 mRNA expression in breast cancer and compare it to that in neighboring normal parental samples. Breast cancer patients exhibited considerably higher LMAN2 mRNA expression levels as opposed to normal samples (*p* < 0.05) (Figures 1(b) and Figures 1(c)). In conclusion, we verified that the LMAN2 mRNA expression is high in diverse tumors using one database and showed substantially elevated LMAN2 expression levels in breast cancer samples using two databases.

### 3.2. LMAN2 expressionis related to clinicopathological characteristics

**Figure 2:**
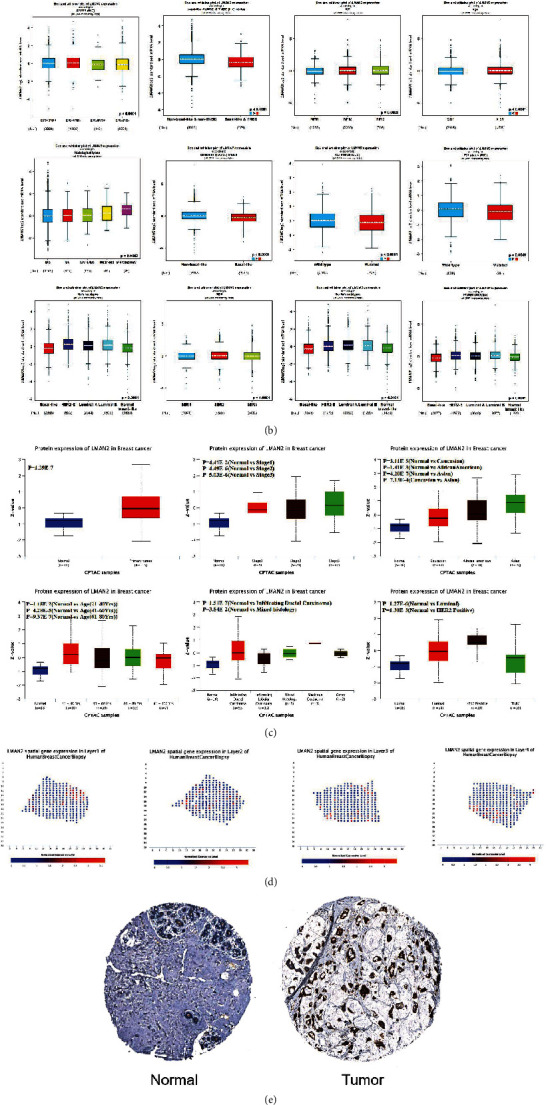
The association between LMAN2 expression and clinicopathological characteristics. (a) The LMAN2 mRNA expression in breast cancer by UALCAN. LMAN2 expression was linked to clinicopathologic characteristics, such as clinical stage, race, sex, p53 mutation status, age, cancer subclasses, distant metastases, and menopause. (b) Analysis of LMAN2 expression in breast cancer using the bc-GenExMiner v4.7 database. Differential expression of LMAN2 in patients with ER-negative and ER-positive, HER2-negative and HER2-positive, lymph node metastasis-positive and lymph node metastasis-negative, p53 mutation-positive and p53 mutation-negative, nontriple negative breast cancer (non-TNBC) and TNBC, nonbasal-like and basal-like, NPI1 vs. NPI2 vs. NPI3, SBR1 vs. SBR2 vs. SBR3, IDC vs. ILC vs. IDC/ILC vs. “mucinous” vs. micropapillary, luminal A vs. HER2-positive vs. luminal B vs. basal-like, and ER-positive/PR-positive vs. ER-negative/PR-positive vs. ER-negative/PR-negative vs. ER-positive/PR-negative breast cancer patients using bc-GenExMiner v4.7. (c) The protein expression of LMAN2 in several subtypes of breast cancer patients as examined with the help of the UALCAN database. Association of LMAN2 expression with clinicopathological characteristics, including clinical stage, race, age, cancer subclasses, histological type, and cancer status. (d) Spatial transcriptomics assessment of gene expression in tissue sections. This work produced high-quality RNA-sequencing data on human breast cancer by importing histologic sections on arrayed reverse transcriptional primers with unique positional barcodes. The data included maintained two-dimensional position data from the histological sections. (e) The protein expression of LMAN2 in breast tumor and normal samples (HPA). (f) The LMAN2 expression in different breast cancer cells by GOBO. (g) The expression of LMAN2 in different breast cancer cells by CCLE and the expression distribution of LMAN2 in different cell lines. The gene expression is shown by the horizontal axis in the figure. The distinct cell lines are represented by the ordinate. The expression level is shown by the dot size and the various colors used in the illustration (^∗∗∗^*p* ≤ 0.001; ^∗∗^*p* ≤ 0.01; ^∗^*p* ≤ 0.05).

A correlation analysis was completed based on data from the UALCAN and bc-GenExMiner v4.7 databases to determine the relationship between LMAN2 expression and clinicopathological parameters. Based on UALCAN database, LMAN2 mRNA expression exhibited a significant positive link to the clinicopathological characteristics, including clinical stage, race, sex, p53 mutation status, age, cancer subclasses, distant metastases, and menopause (*p* < 0.05) (Figure 2(a)). In addition to p53 mutation status, lymph node positivity, and different subtypes, the expression of LMAN2 was shown to have a considerable positive link to some clinicopathological parameters, including ER status, PR status, and HER2+ status (*p* < 0.05) (Figure 2(b)). The protein expression of LMAN2 in several subtypes of breast cancer patients as examined with the help of the UALCAN database. Association of LMAN2 expression with clinicopathological characteristics, including clinical stage, race, age, cancer subclasses, histological type, and cancer status.(Figure 2(c)).We found high expression of LMAN2 in tissue sections by spatial transcriptomics using SpatialDB(Figure 2(d)). Based on HPA, GOBO, and CCLE databases, the results illustrated that LMAN2 protein was expressed in breast cancer and adjoining normal samples (Figure 2(e)) and that a subset of breast cancer cell lines (MDA-MB-157 and MDA-MB-415) had high expression of LMAN2 (Figures 2(f), 2(g)). However, some cell lines (HCC70 and MDA-MB-361) exhibited low expression of LMAN2 (Figures 2(f) and 2(g)). In conclusion, LMAN2 expression was correlated with clinical factors such as p53 mutation status, sex, race, lymph node positivity, and HER2+ classification.

### 3.3. Elevated LMAN2 level in breast canceris associated with a dismal prognosis

**Figure 3:**
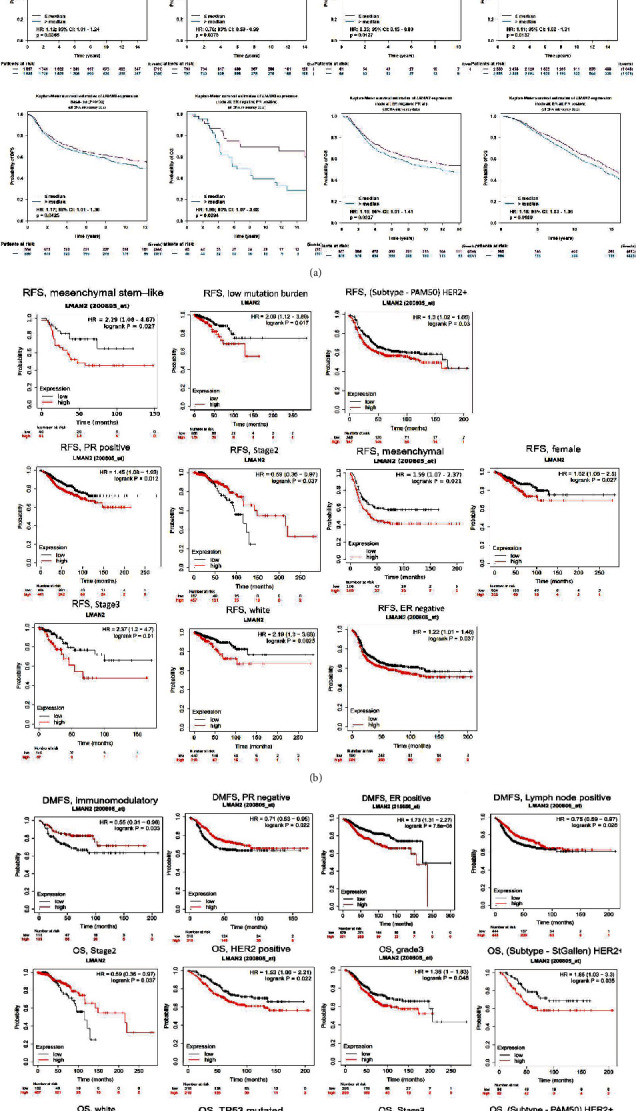
An elevated expression level of LMAN2 in breast cancer is linked to an unfavorable prognosis. Correlation between LMAN2 gene expression with DMFS, RFS, prognosis, and disease-free survival (DFS) in breast cancer patients. The overexpression of LMAN2 was considerably linked to patient survival and resulted in adverse prognostic values. A *p* value < 0.05 represents a significant difference between (a–c) LMAN2 gene and prognosis.

LMAN2 was investigated for its predictive significance in breast cancer utilizing the KM plotter and the bc-GenExMiner v4.7 databases. The prognosis of LMAN2 was found to be related to being female, lymph node positivity, p53 mutation status, stage 2, stage 3, grade 3, HER2+, and white race. The KM plotter and the bc-GenExMiner v4.7 databases demonstrated that elevated LMAN2 expression level was linked to the unfavorable OS, disease-free survival (DFS), and RFS in breast cancer (Figures 3(a)–3(c)). Overall, as a consequence of these findings, LMAN2 expression may be linked to a dismal prognosis among breast cancer patients. Multivariate Cox regression analysis showed that LMAN2 expression and T stage, age, and radiation therapy could be used as independent prognostic factors for OS (Table 1).

### 3.4. LMAN2 expression in distinct cell populations is positively related to DNA repair, apoptosis, and metastasis of breast cancer cells

**Figure 4:**
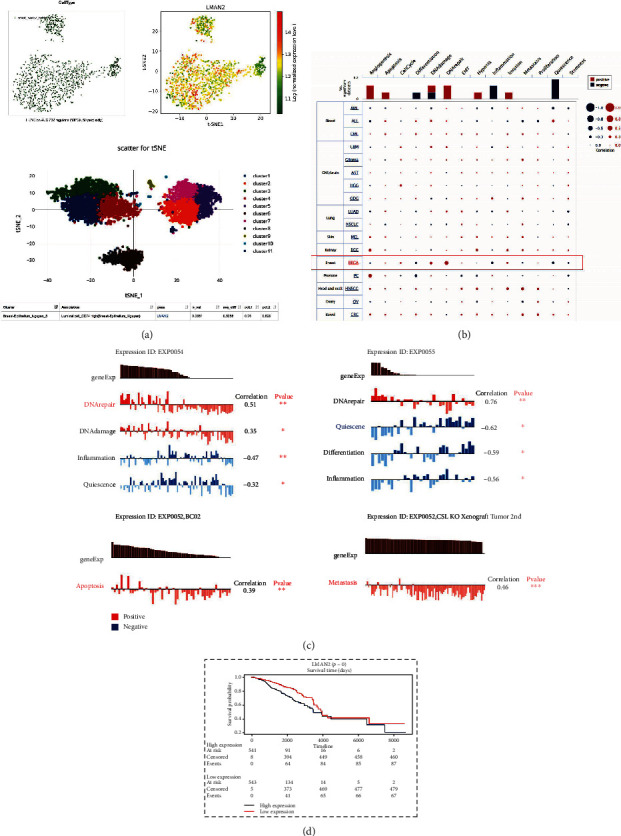
The expression of LMAN2 is, in distinct cellular populations, positively correlated with apoptosis, metastasis, and DNA repair of breast cancer cells. (a) The characteristics of the LMAN2 map and marker genes in breast cancer samples were determined using a human cell landscape. LMAN2 expression in breast-epithelium-Nguyen-8 datasets was statistically significant. A *p* value < 0.05 represents a significant difference. (b) The significance of LMAN2 in 14 functional states of different malignancies, as determined by CancerSEA. (c) Correlation analysis between the LMAN2 expression of functional status in distinct single-cell datasets and functional significance in diverse cell groups using CancerSEA. The correlation of LMAN2 functional status in EXP0052, EXP0054, and EXP0055 single-cell datasets was statistically significant. (d) Single-cell analysis of LMAN2 has a meaningful prognosis (GRNdb) (^∗∗∗^*p* ≤ 0.001; ^∗∗^*p* ≤ 0.01; ^∗^*p* ≤ 0.05).

To acquire a deeper comprehension of the possible function of LMAN2 in breast cancer, further analysis of LMAN2 was undertaken utilizing the Human Cell Landscape database and CancerSEA database. The LMAN2 gene map and the expression characteristics of marker genes in breast cancer samples were compared using the Human Cell Landscape database. LMAN2 is expressed in breast-epithelium-Nguyen-8 ((Basalcel CD74 high, *p* = 0.0061)), and tests are statistically different (Figure 4(a)).

Analysis was conducted with the help of CancerSEA database to compare the correlation of LMAN2 expression in fourteen functional states in different cancers and the correlation of LMAN2 functional states in EXP0052, EXP0054, and EXP0055 single-cell datasets (Figure 4(b)). In EXP0052, LMAN2 expression was statistically different in multiple functional phenotypes, such as metastasis and apoptosis (Figure 4(c) and Table 2). In EXP0054, LMAN2 expression was statistically different in multiple functional phenotypes, such as DNA damage, inflammation, DNA repair, and quiescence (Figure 4(c) and Table 2). In EXP0055, LMAN2 expression was statistically different in multiple functional phenotypes, such as differentiation, inflammation, DNA repair, and quiescence (Figure 4(c) and Table 2). Prognostic analysis showed that elevated LMAN2 expression level was linked to a grim prognosis (Figure 4(d)).

Cancer cell populations may differ greatly in terms of the composition of phenotypically varied breast cancer cell subtypes, representing cells with altered functionality and varied activation statuses. In conclusion, LMAN2 is expressed in distinct cellular populations, and its expression is positively linked to DNA repair, apoptosis, and metastasis of breast cancer cells and is negatively linked to differentiation and inflammation.

### 3.5. LMAN2 expression is associated with homologous recombination (HR) in HER2+ breast cancer

**Figure 5:**
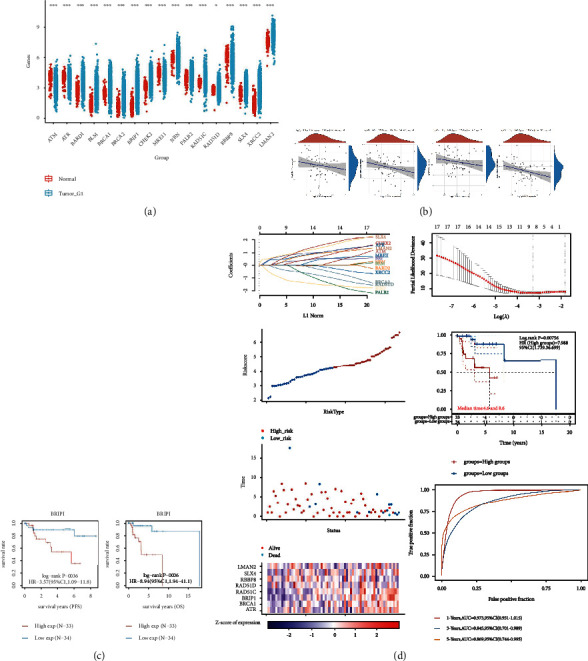
LMAN2 is associated with homologous recombination (HR). (a) HR panel of 16 genes expresses differences in HER2 subtypes. (b) The LMAN2 expression is inversely linked to that of BRCA2, MRE11, and BRIP1. (c) The prognostic analysis shows that BRIP1 has a meaningful prognosis in HER2 subtypes. (d) The HR-associated proteins SLX4, RAD51D, BLM, PALB2, CHEK2, BRIP1, BRCA2, MRE11A, BRCA1, NBN, BARD1, RAD51C, ATR, RBBP8, MRE11, XRCC2, and LMAN2 were used to construct an advanced prognostic model. The lambda parameter displays the coefficients of several characteristics that have been chosen. The LASSO Cox regression model was utilized to plot the partial likelihood deviance vs. log (*λ*). The prognostic analysis of gene signature in TCGA set. The dashed line indicates the median risk score and classifies the patients into 2 groups: those at low risk and those at high risk (^∗∗∗^*p* ≤ 0.001; ^∗∗^*p* ≤ 0.01; ^∗^*p* ≤ 0.05).

Further study of HR was carried out on the basis of TCGA database in order to get a better understanding of the possible involvement of HR in breast cancer. We discovered that the expression characteristics of HR genes in breast cancer samples were significant (Figure 5(a)): X-Ray Repair Cross Complementing 2 (XRCC2), SLX4 structure-specific Endonuclease Subunit (SLX4), RB Binding Protein 8, Endonuclease (RBBP8), BRCA2 DNA Repair Associated (BRCA2), RAD51 Paralog C (RAD51C), MRE11 meiotic recombination 11 homolog A(MRE11A), Checkpoint Kinase 2 (CHEK2), Nibrin (NBN), BRCA1 Interacting Helicase 1 (BRIP1), Partner And Localizer of BRCA2 (PALB2), BRCA1 DNA Repair Associated (BRCA1), RAD51 Paralog D (RAD51D), BLM RecQ Like Helicase (BLM), BRCA1-associated RING domain 1 (BARD1), Ataxia telangiectasia and Rad3 related (ATR), and Ataxia telangiectasia mutated (ATM). The expression of HR-related proteins in HER2 subtypes was different. Correlation analysis illustrated an inverse link between the expression of LMAN2 and that of BRCA2, MRE11, and BRIP1 (Figure 5(b)). Prognostic analysis showed that BRIP1 expression (OS, *p* = 0.006l; PFS, *p* = 0.036) was related to an improved prognosis for HER2+ breast cancer patients (Figure 5(c)). Advanced prognostic model analysis showed that the prognosis of patients with expression of HR DNA damage repair-related proteins, including LMAN2, was intentional (*p* = 0.00756; AUC = 0.973, 0.845, and 0.869 over 1, 3, and 5 years, correspondingly). Therefore, LMAN2 may be a risk factor for HR (Figure 5(d)).

### 3.6. LMAN2 harbors m6A modifications in HER2+ breast cancer

Interestingly, we discovered that LMAN2 was linked to m6A modifications. In the M6A2Target database, the target gene predicted by high-throughput sequencing data analysis includes three parts: validated targets, binding, and perturbation.

The validated target module, the Methyltransferase 3, N6-Adenosine-Methyltransferase Complex Catalytic Subunit (METTL3) modification of LMAN2, was found in human embryonic kidney (HEK-293) cells (Table 3). Through the binding module, in HeLa, HEK293T cells based on crosslinking-immunoprecipitation and high-throughput sequencing (CLIP-Seq) and mass spectrometry technology, we found LMAN2 m6A readers, including Insulin-Like Growth Factor 2 MRNA Binding Protein 1 (IGF2BP1), YTH Domain Containing 1 (YTHDC1), Insulin-Like Growth Factor 2 MRNA Binding Protein 3 (IGF2BP3), and YTH N6-Methyladenosine RNA Binding Protein 1 (YTHDF1) (Table 4), and m6A writers like Vir Like M6A Methyltransferase Associated (VIRMA) (Table 4). Through the perturbation module, in HeLa, A549, Mono-Mac-6, and HepG2 cells based on RNA-Seq, Methylated (m6A) RNA ImmunoPrecipitation with high-throughput Sequencing (MeRIP-Seq), and ribosome profiling technology, we found LMAN2 m6A writers such as Zinc Finger CCCH-Type Containing 13 (ZC3H13), METTL3, VIRMA, Cbl Protooncogene-Like 1 (HAKAI), and WT1-Associated Protein (WTAP) (Table 4), and eraser like FTO Alpha-Ketoglutarate-Dependent Dioxygenase (FTO) (Table 5).

**Figure 6:**
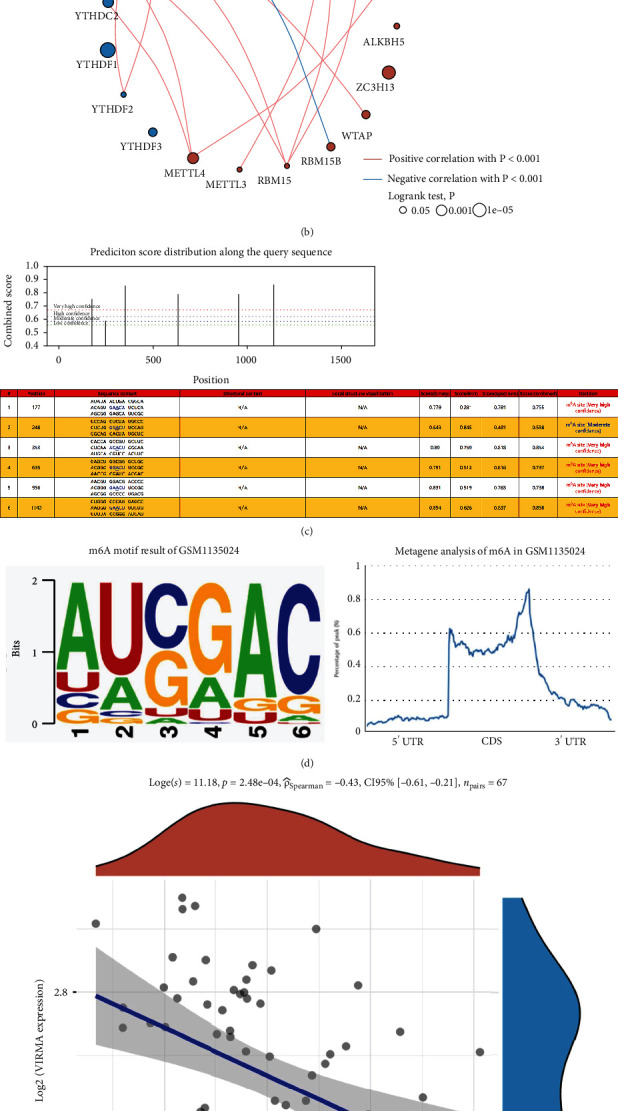
m6A modifications in the LMAN2 gene. (a) Differences in m6A expression in HER2 subtypes. G means tumor group. (b) Correlation analysis of m6A in HER2 subtypes. (c) Prediction of results based on the LMAN2 sequence. (d) *De novo* m6A motif result of GSM1135024. (e) VIRMA is inversely linked to LMAN2 expression. (f) Prognostic analysis of the prognosis of VIRMA and YTHDF1 in HER2 subtypes (^∗∗∗^*p* ≤ 0.001; ^∗∗^*p* ≤ 0.01; ^∗^*p* ≤ 0.05).

First, we examined the m6A protein expression in HER2-positive breast cancer (Figure 6(a)). Following that, we examined the relationships between m6A proteins and observed that METTL3 expression was positively linked to m6A expression (Figure 6(b)). In order to anticipate the m6A alteration domains on the RNA sequences of LMAN2, we employed the SRAMP database. We found that LMAN2 had five m6A domains (extremely high confidence) and one m6A site (moderate confidence) (Figure 6(c)).  Figure 6(d) is the result of the de novo initio m6A motif of GSM1135024. On the basis of the results of the correlation study, VIRMA expression was shown to be inversely linked to LMAN2 expression (Figure 6(e)). The prognostic analysis showed that IGF2BP1 and YTHDF1 had a significant prognosis in the HER2 subtype (Figure 6(f)).

### 3.7. Positive correlation analysis of IC50 score and LMAN2 expression in breast cancer

**Figure 7:**
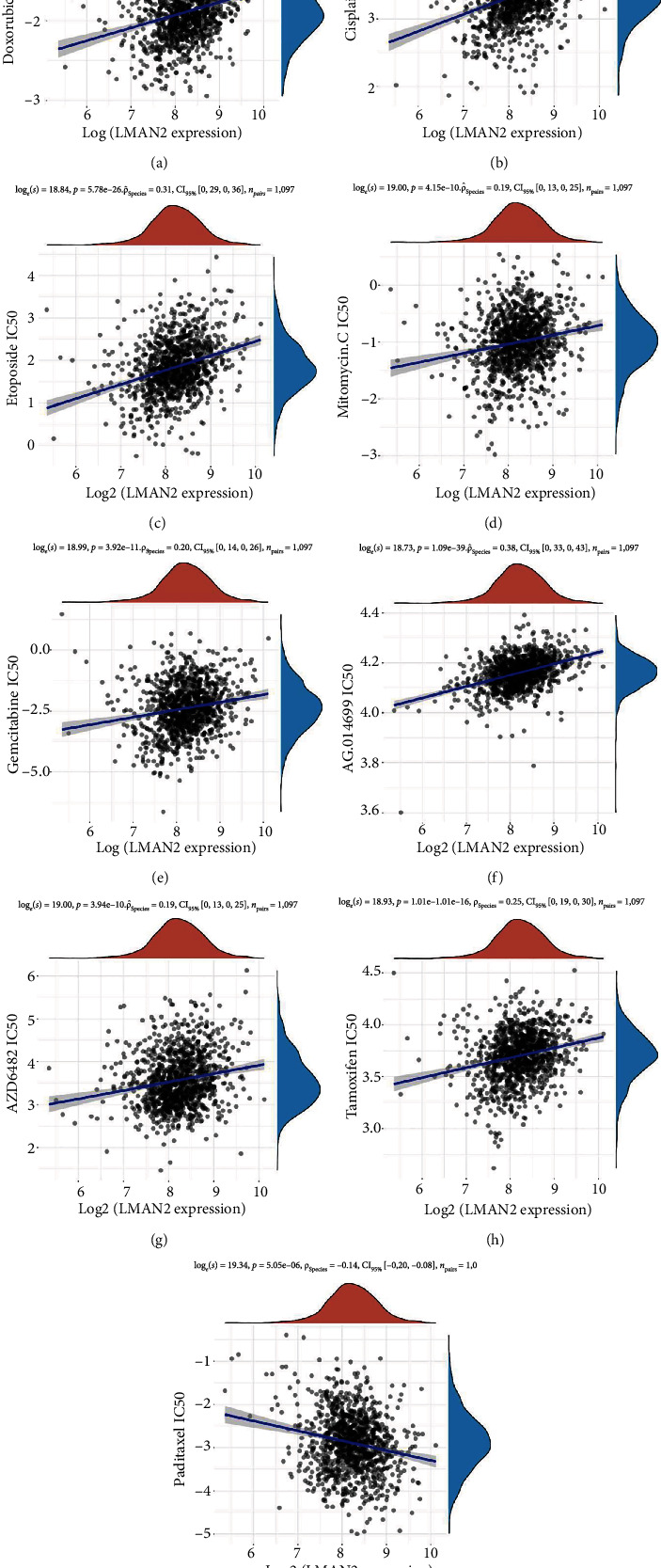
Positive correlation analysis of IC50 score and LMAN2 expression in breast cancer. Pearson's correlation analysis of IC50 score and LMAN2 expression. In the figure (a–i), the horizontal axis represents the LMAN2 expression distribution. The coordinate is the IC50 score distribution. The distribution pattern of IC50 scores is depicted by the density curve on the right. The LMAN2 expression distribution pattern is shown by the density curve in; the topmost value denotes the correlation *p* value, coefficient of correlation, and technique for calculating correlations.

Next, we explored the link between LMAN2 expression in breast cancer and drug sensitivity using GDSC databases and identified a positive link between the LMAN2 expression and the resistance of breast cancer cells to multiple DNA damage chemotherapeutic drugs, such as cisplatin and mitomycin (*p* ≤ 0.05) (Figures 7(b) and 7(d)). In conclusion, LMAN2 expression is positively correlated with resistance to multiple drugs in breast cancer.

### 3.8. LMAN2 is related to DNA damage repair

**Figure 8:**
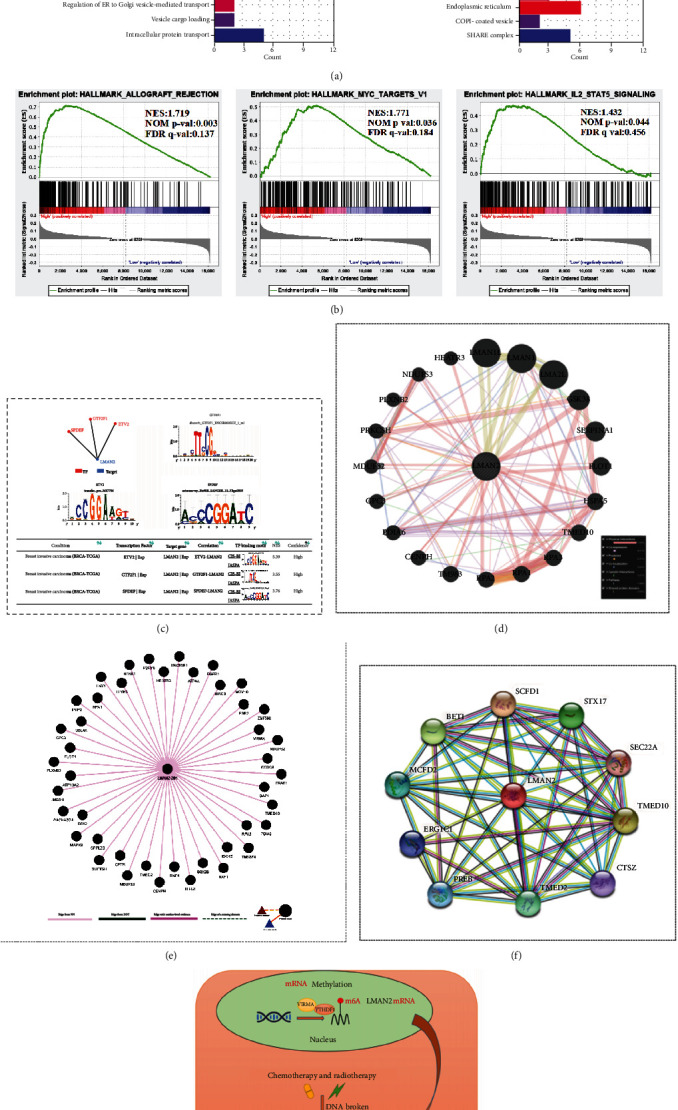
LMAN2 expression is related to DNA damage repair. (a) Gene Ontology GO annotation analyses revealed the functional enrichment of LMAN2. (b) Plots of enrichment derived from GSEA Enrichment Analysis (GSEA). The findings of GSEA showed that ALLOGRAFT_REJECTIOO, IL-2_STAT5_SIGNAL and MYC_TARGETS_V1 were differentially enriched in LMAN2-related enrichment score (ES), normalized *p* value (NOM *p* val), and normalized ES (NES). (c) ETV2, GTF2F1, and SPDEF transcriptional regulation of LMAN2 (GRNdb). (d) Predicted protein-protein interaction network (GeneMANIA). (e) Predicted protein-protein interaction network (DIGGER). (f) Predicted protein-protein interaction network (STRING). (g) m6A methylation modifications of RNA may affect LMAN2 expression, which is associated with homologous recombination (HR) in the DNA damage repair pathway of LMAN2. Predict the mechanistic pattern of LMAN2 in breast cancer.

GO analysis showed that LMAN2 was related to the protein vesicle transport involved in the Golgi apparatus (Figure 8(a)). GSEA analysis revealed a relationship between LMAN2 and myc targets (Figure 8(b)). CIS-BP and JASPAR analyses showed that LMAN2 was transcriptionally regulated by ETS Variant Transcription Factor 2 (ETV2),SAM Pointed Domain Containing ETS Transcription Factor(SPDEF), and General Transcription Factor IIF Subunit 1 (GTF2F1) (Figure 8(c)). LMAN2 is related to Replication Protein A1 (RPA1), Replication Protein A2 (RPA2), Replication Protein A3 (RPA3), and other DNA damage repair proteins, as determined by protein-protein interaction analyses utilizing GeneMANIA, DIGGER, and STRING databases (Figures 8(d)–8(f)).(Figure 8(g)). Therefore, based on the single-cell level analysis of LMAN2, drug resistance analysis, DNA damage repair analysis, and protein-protein network analysis results, we hypothesize that LMAN2 is involved in DNA damage repair.

## 4. Discussion

In the last several years, significant advancements have been achieved in the management of HER2-positive breast cancer. These advancements have prolonged patients' survival duration and have established themselves as essential therapeutic options for HER2-positive advanced breast cancer. Targeted therapy still has the risk of cardiotoxicity, low single-drug effective rate, heterogeneous efficacy, and high price. At the same time, due to the few clinical studies of new drugs, short application time, and limited data for second-line and higher-level treatments, many treatment options remain controversial [37, 38].

Considering the age of patients, tumor size, the metastatic times of the axillary lymph nodes, and the histopathological grades, there are differences in the expression of HER2, PR, and ER. Therefore, it is important to comprehensively evaluate HER2 expression and develop personalized treatment strategies.

In this research, we began by performing an integrated analysis of LMAN2 expression using several omics databases. Multiple databases verified that LMAN2 is expressed at a high level in breast cancer cells, and this expression is linked to an unfavorable clinical prognosis of HER2-positive+ breast cancer. Additionally, we analyzed the high expression and poor prognosis of LMAN2 at mRNA, and protein levels, as well as at spatial transcriptome and cellular levels. The combined analysis showed that LMAN2 expression is correlated with p53 mutation status, age, sex, race, lymph node metastasis, and tissue type. Second, multiple prognostic databases showed that an elevated level of LMAN2 was linked to adverse prognosis among HER2-positive breast cancer patients, and that the elevated level of luminal A type was linked to a good prognosis. Additionally, the prognosis of LMAN2 is related to sex, race, lymph node metastasis, p53 mutations, stage, and HER2 positivity.

Similar to DNA or histone modification, m6A modification is an epigenetic modification. Through the cocatalytic regulation of m6A methyltransferase and demethylase, m6A modification participates in diverse biological functions, including RNA splicing, protein translation, and stem cell regeneration [39, 40]. In gastrointestinal cancer, the m6A RNA alteration has an effect on the PI3K/Akt/mTOR signaling pathways [41]. In breast cancer, the expression of m6A RNA methylation has been shown to have clinical prognostic significance [42].

In this study,we evaluated the m6A-related protein expression in the HER2 subtype. A joint analysis of multiple m6A databases showed that LMAN2 had m6A modifications, and that there were high-scoring m6A modifications in multiple sequences. LMAN2 expression is negatively correlated with the m6A writer VIRMA expression. The expression of the m6A readers YTHDF1 and IGF2BP1 was linked to a grim prognosis in HER2 subtypes. Antagonists of m6A-related factors, have been found, and some of them exhibit the potential to suppress cancer progression, suggesting that m6A could potentially serve as a therapeutic target for cancer. Consequently, the m6A alteration in the LMAN2 gene could provide a promising therapeutic target for treating HER2-positive breast cancer.

Breast cancer is a malignant illness that manifests itself in a variety of ways. Single-cell RNA-Seq can specifically identify a certain type of cell and its corresponding gene expression characteristics in the tumor microenvironment [43]. In addition to specific descriptions of certain types of immune cell characteristics, single-cell RNA-Seq data can provide information on the cell composition and distribution characteristics of the tumor immune microenvironment from a holistic perspective [44]. Analysis of multiple datasets in the single-cell database shows that LMAN2 is positively correlated with DNA damage repair, metastasis, and apoptosis. LMAN2 expression is negatively correlated with differentiation and inflammation. Meanwhile, we used different datasets to evaluate the possible functions of LMAN2 in breast cancer. The findings revealed that LMAN2 expression varies depending on the tumor microenvironment of breast cancer. The aforementioned single-cell result analysis suggests that single-cell RNA-Seq may be utilized to analyze the tumor immune microenvironment at a higher resolution level, accurately characterize its various cell groups and related transcriptional features, discover new clinical immunotherapeutic targets, and analyze the prognosis for individuals with various kinds of malignancies in terms of survival [45].

According to the clinical practice guidelines for systemic treatment of HER2-positive advanced breast cancer and the principle of treating HER2-positive advanced breast cancer, the selected therapeutic plan recommends targeted therapy combined with chemotherapy and endocrine therapy. However, chemotherapy and endocrine resistance are the reasons for the poor outcomes in HER2-positive breast cancer therapy. In this research, LMAN2 is resistant to multiple DNA-damaging chemotherapeutics and endocrine drugs, such as anthracyclines, platinum, and tamoxifen. However, LMAN2 is sensitive to the chemotherapy drug paclitaxel. The above analysis suggests that HER2-positive breast cancer patients can be treated with paclitaxel.

The random energy deposition of infrared radiation (IR) can result in multiple DNA damages, such as single-strand breaks, double-strand break (DSB), and various types of base damage, including thymine glycol [46]. DSBs are by far the most genotoxic of all DNA damages, and they are induced by ionization of clusters due to a single radiation orbit, leading to tightly spaced single-strand breaks at a single or several injury sites [46]. ssDNA breaks and base damages induced by IR have the potential to impair the replication of DNA and result in a unilateral DSB. Endogenous chemical substances and exogenous environmental factors can continue to threaten the stability of genetic material, resulting in various DNA damages. These damages may come under the effect of intracellular and extracellular physical and chemical factors such as ultraviolet rays, ionizing radiation, toxic reagents, and reactive-oxygen free radicals. If these damages are not repaired on time, they may interfere with normal cellular functions. For example, damage to key genes such as tumor suppressors will greatly increase the possibility of tumor development [47]. Fortunately, biological cells have evolved, and DNA repair pathways are in place to remove these damages. DSB, the most serious type of DNA damage, mainly includes three pathways competing for the repair of DSBs such as nonhomologous end-joining (NHEJ) and HR [48, 49]. HR is a highly accurate DNA repair mechanism that mainly relies on homologous chromosomes to guide the correction of damaged DNA, while nonhomologous end-joining directly connects the two broken DNA strands together [50]. The key proteins in HR, BRCA1, and BRCA2 are two important tumor suppressors. In the absence of these two proteins, the rate of homologous recombination in the cell will be greatly reduced, thus rendering the cell sensitive to ionizing radiation. Normal cells can recover from DNA damage through HR and survive. However, tumor cells can restore the DNA damage induced by chemotherapeutic drugs through HR, thereby promoting drug resistance. HR recognizes DNA DSBs through the MRE11-RAD50-NBS1 protein complex (MRN complex), which has a variety of catalytic enzyme functions for processing and sequencing DNA ends [51]. In addition, BRCA2 protects DNA by stabilizing RAD51 Recombinase (RAD51) filaments. The stalled replication forks are protected from extensive nuclear lysis and degradation [52]. Upon DNA damage, ATM and other members related to DNA damage repair are activated, and through phosphorylation of the corresponding downstream proteins, they regulate the process of the cell cycle and promote DNA damage repair, thereby playing a vital function in the maintenance of genome stability [53].

In this research, we initially analyzed the differences in the expression of HR-related proteins that repair DNA damage in HER2 subtypes. The LMAN2 expression level was inversely linked to BRCA2, MRE11, and BRIP1. In terms of OS and PFS, BRIP1 expression led to a significant prognosis in the HER2 subtypes. Breast cancer patients exhibiting LMAN2 expression have a dismal prognosis, according to the findings from an advanced prognostic model analysis. On the other hand, the LMAN2 expression was shown to have a positive link to the expression of DNA damage repair-associated proteins, according to single-cell analysis. The drug resistance analysis showed that LMAN2 expression rendered the breast cancer cells resistant to a variety of DNA damage-inducing chemotherapeutic drugs, such as anthracyclines or platinum. Single-cell analysis and drug resistance analyses combined with DNA damage repair analysis showed that LMAN2 might be involved in HR DNA damage repair, thereby affecting the chemotherapy resistance of HER2 subtype breast cancer, consequently affecting the prognosis. Therefore, LMAN2 may be a new target of HR that contributes to the development of HER2 subtype-targeting anticancer drugs. The proteins encoded by LMAN2 have been shown to bind to glycoproteins of the high mannose type, and this promotes their quality control, trafficking, and sorting. Some of its related pathways include the metabolism of proteins, transportation to the Golgi, and consequent modification. Previous studies have shown that LMAN2 is a candidate tumor biomarker in ovarian cancer [10]. LMAN2 regulates the transport of exosomal cargo proteins through the Golgi complex [11]. LMAN2 regulates the trafficking of GPRC5B, an exosomal cargo protein, from the trans-Golgi network (TGN) to the endosomes for the purpose of facilitating exosome secretion [54]. LMAN2 is a HUB gene resistant to cisplatin in gastric cancer [55]. However, the expression, prognosis, and function of LMAN2 in other tumors are still unclear, especially in breast cancer. In this study, we utilized various databases to investigate the expression, prognosis, and possible function of the LMAN2 gene. Our comprehensive analysis using multiple databases shows that LMAN2 may influence breast cancer patients' prognoses by affecting expression via m6A methylation, and DNA damage repair. The outcomes of this research might serve as a foundation for improving the detection and treatment of breast cancer in clinical setting. There are certain limitations to this study. For example, the m6A modification status and potential function of LMAN2 have not been further verified through *in vivo* and *in vitro* studies. In the future, we plan to verify that LMAN2 is involved in the HR-specific process of DNA damage repair in breast cancer through cell and animal experiments and explore the role that LMAN2 plays in HR. We anticipate that our findings will contribute to the discovery of molecular targets for breast cancerdiagnosis and treatment.

## Figures and Tables

**Table 1:** Univariate and multivariate analyses of factors associated with LMAN2 survival.

Characteristics	Total (*N*)	Univariate analysis		Multivariate analysis	
		Hazard ratio (95% CI)	*p* value	Hazard ratio (95% CI)	*p* value
T stage	1079				
T1 & T2	905	Reference			
T3 & T4	174	1.608 (1.110-2.329)	0.012	2.361 (1.081-5.157)	0.031
N stage	1063				
N0 & N1	871	Reference			
N2 & N3	192	2.163 (1.472-3.180)	<0.001	1.614 (0.570-4.573)	0.367
M stage	922				
M1	20	Reference			
M0	902	0.235 (0.136-0.405)	<0.001	0.390 (0.120-1.269)	0.118
LMAN2	1082	1.009 (0.779-1.308)	0.944		
Race	993				
White	753	Reference			
Asian	60	0.754 (0.239-2.383)	0.631		
Black or African American	180	1.151 (0.765-1.731)	0.501		
Age	1082				
≤60	601	Reference			
>60	481	2.020 (1.465-2.784)	<0.001	3.810 (2.028-7.159)	<0.001
PR status	1029				
Negative	342	Reference			
Positive	687	0.732 (0.523-1.024)	0.068	0.756 (0.311-1.842)	0.539
ER status	1032				
Negative	240	Reference			
Positive	792	0.712 (0.495-1.023)	0.066	0.486 (0.190-1.243)	0.132
HER2 status	715				
Negative	558	Reference			
Positive	157	1.593 (0.973-2.609)	0.064	0.786 (0.371-1.664)	0.529
Pathologic stage	1059				
Stage I & stage II	799	Reference			
Stage III & stage IV	260	2.391 (1.703-3.355)	<0.001	2.760 (0.859-8.862)	0.088
Radiation therapy	986				
No	434	Reference			
Yes	552	0.576 (0.394-0.841)	0.004	0.475 (0.249-0.904)	0.023

**Table 2:** Analysis of the potential functions of LMAN2 based on CancerSEA database.

CancerSEA	Potential function	Correlation	*p*
EXP0052	Metastasis	0.46	*p* ≤ 0.001
EXP0052	Apoptosis	0.39	*p* ≤ 0.01
EXP0054	DNA damage	0.35	*p* ≤ 0.05
EXP0054	Inflammation	-0.47	*p* ≤ 0.01
EXP0054	DNA repair	0.51	*p* ≤ 0.01
EXP0054	Quiescence	-0.32	*p* ≤ 0.05
EXP0055	Differentiation	-0.59	*p* ≤ 0.05
EXP0055	Inflammation	-0.56	*p* ≤ 0.05
EXP0055	DNA repair	0.76	*p* ≤ 0.01
EXP0055	Quiescence	-0.62	*p* ≤ 0.05

**Table 3:** The experiment validated m6A target genes.

Validated targets					
Species	Cell line	Class	WER name	Target gene	PMID
Human	HEK293	Writer	METTL3	LMAN2	29924987

WERs: writers, erasers, and readers.

**Table 4:** The target genes that inferred from CLIP-Seq, RIP-Seq, CHIP-Seq, or mass spectrometry.

Binding							
Species	Cell line	Class	WER name	Target gene	Interaction	Method	Downstream effect
Human	HeLa	Writer	VIRMA	LMAN2	Protein-protein	Mass spectrometry	Methylation
Human	HEK293T	Reader	IGF2BP1	LMAN2	Protein-RNA	CLIP-Seq	No evidence
Human	HEK293T	Reader	IGF2BP3	LMAN2	Protein-RNA	CLIP-Seq	No evidence
Human	HEK293T	Reader	YTHDC1	LMAN2	Protein-RNA	CLIP-Seq	No evidence
Human	HEK293T	Reader	YTHDF1	LMAN2	Protein-RNA	CLIP-Seq	No evidence
Human	HeLa	Writer	VIRMA	LMAN2	Protein-protein	Mass spectrometry	Methylation
Human	HEK293T	Reader	IGF2BP1	LMAN2	Protein-RNA	CLIP-Seq	No evidence
Human	HEK293T	Reader	IGF2BP3	LMAN2	Protein-RNA	CLIP-Seq	No evidence
Human	HEK293T	Reader	YTHDC1	LMAN2	Protein-RNA	CLIP-Seq	No evidence
Human	HEK293T	Reader	YTHDF1	LMAN2	Protein-RNA	CLIP-Seq	No evidence
Human	HeLa	Writer	VIRMA	LMAN2	Protein-protein	Mass spectrometry	Methylation

WERs: writers, erasers, and readers. “Protein-RNA” represents the genes inferred from various kinds of CLIP-Seq or RIP-Seq. “Protein-DNA” refers to the CHIP-Seq results, while “protein-protein” means targets summarized from mass spectroscopy results.

**Table 5:** The differential expression, differential translation efficiency, or differential methylation genes upon perturbation of WERs.

Perturbation						
Species	Cell line	Class	WER name	Target gene	Interaction	Downstream effect
Human	HeLa	Writer	VIRMA	LMAN2	Protein-protein	Methylation
Human	HeLa	Writer	HAKAI	LMAN2	No evidence	Expression
Human	HepG2	Writer	METTL3	LMAN2	No evidence	Expression
Human	HeLa	Writer	WTAP	LMAN2	No evidence	Methylation
Human	A549	Writer	VIRMA	LMAN2	No evidence	Methylation
Human	A549	Writer	WTAP	LMAN2	No evidence	Methylation
Human	Mono-Mac-6	Eraser	FTO	LMAN2	No evidence	Methylation
Human	HeLa	Writer	ZC3H13	LMAN2	No evidence	Methylation
Human	HeLa	Writer	METTL3	LMAN2	No evidence	Methylation; translation
Human	HeLa	Writer	VIRMA	LMAN2	Protein-protein	Methylation
Human	HeLa	Writer	HAKAI	LMAN2	No evidence	Expression
Human	HepG2	Writer	METTL3	LMAN2	No evidence	Expression
Human	HeLa	Writer	WTAP	LMAN2	No evidence	Methylation
Human	A549	Writer	VIRMA	LMAN2	No evidence	Methylation

WERs: writers, erasers, and readers. “Protein-protein” means targets summarized from mass spectroscopy results.

## Data Availability

The data used to support the findings of this study are included within the article.
